# Mitochondrial Degeneration and Autophagy Associated With Delayed Effects of Radiation in the Mouse Brain

**DOI:** 10.3389/fnagi.2019.00357

**Published:** 2019-12-20

**Authors:** Neel K. Sharma, Sasha Stone, Vidya P. Kumar, Shukla Biswas, Saeed Y. Aghdam, Gregory P. Holmes-Hampton, Christine M. Fam, George N. Cox, Sanchita P. Ghosh

**Affiliations:** ^1^Armed Forces Radiobiology Research Institute, Uniformed Services University of the Health Sciences, Bethesda, MD, United States; ^2^Bolder Biotechnology, Inc., Boulder, CO, United States

**Keywords:** radiation countermeasure, BBT-059, DEARE, DRF, mitochondria, autophagy

## Abstract

Mitochondria are linked with various radiation responses, including mitophagy, genomic instability, apoptosis, and the bystander effect. Mitochondria play an important role in preserving cellular homeostasis during stress responses, and dysfunction in mitochondrial contributes to aging, carcinogenesis and neurologic diseases. In this study, we have investigated the mitochondrial degeneration and autophagy in the hippocampal region of brains from mice administered with BBT-059, a long-acting interleukin-11 analog, or its formulation buffer 24 h prior to irradiation at different radiation doses collected at 6 and 12 months post-irradiation. The results demonstrated a higher number of degenerating mitochondria in 12 Gy BBT-059 treated mice after 6 months and 11.5 Gy BBT-059 treated mice after 12 months as compared to the age-matched naïve (non-irradiated control animals). Apg5l, Lc3b and Sqstm1 markers were used to analyze the autophagy in the brain, however only the Sqstm1 marker exhibited significantly reduced expression after 12 months in 11.5 Gy BBT-059 treated mice as compared to naïve. Immunohistochemistry (IHC) results of Bcl2 also demonstrated a decrease in expression after 12 months in 11.5 Gy BBT-059 treated mice as compared to other groups. In conclusion, our results demonstrated that higher doses of ionizing radiation (IR) can cause persistent upregulation of mitochondrial degeneration. Reduced levels of Sqstm1 and Bcl2 can lead to intensive autophagy which can lead to degradation of cellular structure.

## Introduction

The impact of ionizing radiation (IR) on the central nervous system (CNS) was first reported more than a decade ago (Valentin, 2005), recently there has been increasing interest to further comprehend these effects. In this world, the universal threat of radiation exposure from war or accidental exposure, the impact of high and low doses of IR on the neurobiological tissue and responses to living things needs to be re-evaluated. Hippocampus in the brain is widely affected by IR and is the radio-sensitive region of the brain which hosts proliferating neuronal progenitor cells (Harada et al., 2014; Pospisil et al., 2015). It has been revealed that differentiating cells incorporated into the hippocampal network which leads to apoptosis or dysfunction following exposure to high doses of radiation, which leads to mitochondrial degeneration (Wang, 2001). It was reported that after a high dose of IR, cytochrome c is released by mitochondria to induce apoptosis in irradiated cells (Wang, 2001). Therefore, mitochondria control the death and survival of cells after IR (Shimura and Kunugita, 2016).

Recently we identified a promising radiation countermeasure, a pegylated construct of the 20 kDa protein IL-11 (BBT-059, Bolder Biotechnology, Boulder, CO, USA). IL-11 was isolated in 1990 (Paul et al., 1990), and recombinant human IL-11 (rhIL-11) was developed and marketed to reduce chemotherapy-induced thrombocytopenia but its clinical use is limited by the requirement of multiple daily injections and severe adverse effects in humans (Hauer-Jensen, 2014). To overcome this problem, Bolder Biotechnology has developed BBT-059 (Lee et al., 2012; Plett et al., 2014) which has a longer half-life in plasma of rodents and improved the induction time of hematopoietic stem cells compared to rhIL-11 (Plett et al., 2014). A single dose of BBT-059 led to a stimulation of circulating platelets in rats and mice that lasted up to 10 days (Lee et al., 2012).

Previously, we have demonstrated that BBT-059 is effective as a radiation countermeasure when administered 24 h post-total body irradiation (TBI) as well as when administered 24 h pre-TBI in male CD2F1 mice (Kumar et al., 2018). BBT-059 protects the hematopoietic system and attenuates radiation-induced upregulation of hematopoietic cytokine (Epo, TPO, Flt3L) expression, thereby preventing lethal radiation injury as determined in 30-day survival studies (Kumar et al., 2018). Animals surviving exposure to lethal doses of radiation might experience delayed effects of acute radiation exposure (DEARE) which may manifest after recovery from acute radiation syndrome (Williams et al., 2010) and contribute to an increased incidence of many circulatories, age-related, and neurodegenerative diseases (Sharma et al., 2018). Many neurodegenerative diseases are related to mitochondrial dysfunction and DEARE may disrupt mitochondrial function through several mechanisms. Mitochondria are the richest source of reactive oxygen species (ROS) due to their higher consumption of oxygen and they occupy a fairly substantial fraction of cell volume (Cadenas and Davies, 2000; Leach et al., 2001) of 4–25% depending on the cell, which renders them a likely target of radiation traversal through the cell.

In this study, our aim was to analyze the DEARE on brain mitochondrial degeneration in mice administered with BBT-059 or its formulation buffer. Mitochondria are known to be the death-promoting organelles and are the powerhouse of the cells. Mitochondria are a major site of ROS production and discharge pro-death molecules in response to IR, which results in cell death cascades due to impaired ATP synthesis. Mitochondrial dysfunction has been associated with many human diseases and mutations in mitochondria-encoded proteins cause rare mitochondrial diseases. Mitochondrial-mediated ATP deprivation, impaired cell signaling and oxidative stress have been linked to the pathogenesis of neurodegenerative and metabolic diseases [e.g., Parkinson’s disease (PD), Alzheimer’s disease (AD), and amyotrophic lateral sclerosis (ALS; Lou et al., 2019)]. However, cells have developed a system for clearing aberrant mitochondria and organelles that might cause this damage within cells, a process termed as “autophagy.” Mitochondrial dysfunction changes cellular respiration, energy metabolism and plays a major role in neurodegenerative diseases. Dysfunctional mitochondria may be considered early features of neurodegenerative diseases (Lezi and Swerdlow, 2012). Mitochondrial homeostasis is maintained by mitophagy; once mitophagy is initiated, autophagy will be activated to remove deceased mitochondria and other organelles by lysosomes in normal functioning neurons (Wang et al., 2019). Autophagy is an intracellular degradation system which delivers cytoplasmic constituents to the lysosome, and any disruption in autophagy has been related to increased genome instability. However, damage due to radiation in the brain stem and progenitor cells can cause mitochondrial dysfunction and autophagy which has been shown to be involved in cell death. Autophagy is also a critical regulator of mitochondrial homeostasis. Dysfunctional mitochondria release toxic apoptotic mediators and ROS due to loss in their membrane potential are apparently removed by autophagy process relative to normal mitochondria *via* phosphorylation reactions mediated by the kinase PINK1 and subsequent ubiquitination of mitochondrial membrane proteins by the E3 ligase Parkin (Rubinsztein et al., 2011). The purpose of this study was to evaluate the effect of radiation on mitochondrial degeneration and autophagy in the long term after radiation and pre-treatment with BBT-059, a promising radiation countermeasure, or its formulation buffer. BBT-059 treatment allowed mice to survive exposure to radiation doses of 10.5–12 Gy, which are supra-lethal doses as compared to untreated mice cannot survive at these high radiation doses. Therefore, untreated survivors from 10 Gy whole-body radiation exposure were used as control in this study.

## Materials and Methods

### Animals

The study was carried out in accordance with the recommendations in the Guide for the Care and Use of Laboratory Animals of the National Institutes of Health. This study received approval from the institutional animal care and use committee at the Armed Forces Radiobiology Research Institute (AFRRI). Male CD2F1 mice (8–10 weeks old) were purchased from Envigo, USA. The mice were housed in the AFRRI vivarium, which is accredited by the association for assessment and accreditation of Laboratory Animal Care-International. Experimental animals were housed 4 per box in sterile cages and were identified by a tail tattoo. Before the start of the study, all animals were acclimatized for 1–2 weeks. Animals are fed (Harlan Teklad Global Rodent Diet 8604) and provided acidified water (pH ~2.5) *ad libitum*. The animal rooms were maintained at 21 ± 2°C and 50 ± 10% relative humidity with 10–15 cycles of fresh air hourly and a 12:12 h light: dark cycle.

### Study Design

Ten to 12 weeks old male CD2F1 animals were divided into five groups [naïve, formulation buffer (FB) 10 Gy, BBT-059 10.5 Gy, BBT-059 11.5 Gy and BBT-059 12 Gy]. BBT-059 in FB (0.3 mg/kg) or FB (10 mM sodium phosphate, 4% mannitol, 1% sucrose, pH 6.2) were injected as a single dose (0.1 ml) 24 h prior to TBI at the nape of the neck subcutaneously (SC) before exposure to the specified radiation doses. An estimated dose rate of 0.6 Gy/min in the AFRRI Cobalt-60 γ-irradiation facility was given to mice bilaterally at the Armed Forces Radiobiology Research Institute, Bethesda, MD, USA (Kumar et al., 2018). This study design is a part of determining dose reduction factor (DRF) study of BBT-059 (unpublished data); DRF was determined from radiation dose corresponding to median survival on day 30 post-TBI and the surviving animals from this study were monitored up to 12 months post-TBI to study DEARE. Surviving animals were sacrificed after euthanasia at six and twelve-month post-TBI to collect brains and other major organs (bone marrow, kidney, heart, lung, and eyes). In this study, we analyzed the brains to evaluate the effect of radiation on mitochondrial degeneration and autophagy in long term survivors post-TBI. Survival curve and analysis from other major organs with data have been communicated in a separate manuscript.

### Brain Removal and Fixation

After euthanizing the animals at 6 months and 12 months post-TBI, the skull was cut with scissors and the brain was removed as quickly as possible using a spatula. Brains were placed on a stainless steel brain matrix and sagittal sections were cut. From center towards left, a 2 mm section was used for electron microscopy, 2 mm section was used for immunohistochemistry (IHC) and the rest left part of the brain was used for western blots. These experiments were conducted using three independent animals from each group.

### Electron Microscopy

Samples were fixed overnight at room temperature in 2% formaldehyde which is freshly prepared from paraformaldehyde (PFA) crystals and 2% EM grade glutaraldehyde (Electron Microscopy Sciences, Hatfield, PA, USA) in 0.1 M cacodylate buffer, pH 7.2. Following three washes in the same cacodylate buffer (without aldehydes), samples were incubated in 2% OsO4 in 0.1 M cacodylate buffer (0.1 M, pH 7.2) for 1 h and then washed 3 × 10 min in cacodylate buffer. The CA1 region of the hippocampus was separated using a dissection microscope. Samples were dehydrated in a graduated series of ethanol in water (1 × 10 min each in 30%, 50%, 70%, and 95% ethanol and 2 × 10 min in 100% ethanol). Samples were infiltrated in a graduated series of Spurr’s epoxy resin (Electron Microscopy Sciences, Hatfield, PA, USA) after dehydration, and then polymerized at 70°C for 11 h. Leica UC6 ultramicrotome was used to section thin polymerized blocks and thin sections were collected on 3 mm copper grids. Leica EM AC20 was used to post-stained the grids and then examined on a JEOL JEM-1011 transmission electron microscope (JEOL USA, Peabody, MA, USA). Images were taken on an Advanced Microscopy Techniques (AMT Corporation, Woburn, MA, USA) digital camera. To characterize the degree of mitochondrial degeneration, the numbers of degenerating mitochondria were counted in four randomly selected, nonoverlapping fields of the CA1 region above the hippocampal sulcus from each animal.

### Western Blot Analysis

At 6 months and 12 months, post-TBI a part of left-brain sections were isolated as discussed above. The sections were snap-frozen and stored at −80°C. The tissue was homogenized and lysed in ice-cold RIPA buffer (Sc24948A Santa Cruz Biotechnology, Santa Cruz, CA, USA) in the presence of protease inhibitors (Sigma P8340). The homogenates were centrifuged at 12,000 × g for 10 min at 4°C, and the total protein concentrations were determined using a BCA protein assay (Thermo Fisher). After normalizing the concentrations, 4× Laemmli sample buffer (BIORAD #1610747) was added to achieve a 1× final concentration. A reducing agent (2-mercaptoethanol) was added to the buffer prior to mixing with the sample. Samples were denatured by boiling at 100°C for 5 min and were run on 4–12% SDS-PAGE gels at 80 volts for 60 min and then transferred to PVDF membranes by semi-dry transfer (Trans-Blot^®^ Turbo^TM^ Transfer System, BIORAD). Membranes were blocked with 4% bovine serum albumin (BSA) in 1× TBST (tris buffered saline with tween20) and immunoblots were incubated with either rabbit monoclonal anti-recombinant APG5L/ATG5 antibody (ab109490, Abcam), rabbit polyclonal anti-LC3B antibody (ab48394, Abcam), and rabbit monoclonal anti-recombinant SQSTM1/p62 antibody (ab109012, Abcam) at 4°C overnight with gentle agitation. Membranes were then washed three times with TBST and incubated with horseradish peroxidase-conjugated secondary antibody (Invitrogen #31460) for 60 min. Membranes were washed three times with TBST and then incubated with enhanced chemiluminescence reagents (GE Healthcare RPN2106) and finally developed using (ChemiDoc^TM^ Imaging Systems, BIORAD). A mouse monoclonal antibody against β-actin (Santa Cruz Sc47778) was used as a loading control. The intensities of specific bands corresponding to the proteins of interest were measured by using ImageJ software.

#### Immunohistochemistry

For tissue sections, brain samples were prepared by fixing with 4% PFA at room temperature, followed by washing with 1× PBS. All the samples were kept in 10% sucrose for 2 h, 20% sucrose overnight at 4°C, and then in 30% sucrose for 4 h at room temperature. One percentage BSA solution was used for blocking the samples for 30 min and then embedded in Tissue-Tek^®^ OCT^TM^ Compound (Sakura Finetek Europe B.V., Netherlands) and 11 μm sections prepared. The samples were incubated with primary antibodies (Bcl2, Cell Signaling #3498) at a concentration of 1:100 overnight at 4°C and with secondary antibody (1:500) for 2 h at room temperature. Confocal images were obtained using a Zeiss 700 camera.

## Results

### Delayed Effects of Acute Radiation Exposure Cause Mitochondrial Degeneration

**Figure 1:**
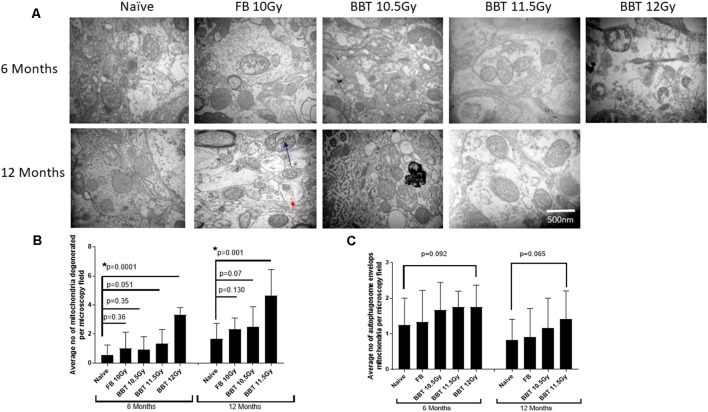
Higher radiation dose leads to mitochondrial degeneration as delayed effects.** (A)** Hippocampus from naive, animals treated with formulation buffer and BBT-059 were collected at 6 and 12 months post-total body irradiation (TBI). The figure shows the representative images of electron microscopy from the hippocampus at 6 and 12 months from naive, 10 Gy irradiated pre-treated with formulation buffer (FB 10 Gy), 10.5, 11.5, and 12 Gy irradiated and pre-treated with BBT-059. **(B)** The bar graph shows the average number of degenerating mitochondria from 12 pictures at 60,000× from different regions of three independent animals (Scale bar 500 nm) at 6 and 12 months. After 6 months the number of degenerating mitochondria were significantly higher in 12 Gy BBT-059 treated group as compared to naïve (*p* = 0.0001), however, after 12 months the number of degenerating mitochondria in 11.5 Gy BBT-059 treated group were significantly high as compared to naïve (*p* = 0.001). Black arrow is pointing to the degenerating mitochondria. **(C)** No significant difference was observed in autophagosome envelops measured from electron microscopy pictures. Red arrow is pointing to the autophagosome envelope. Mann–Whitney *U* test was applied for analysis. *p < 0.05.

For this study, we have performed transmission electron microscopic technique (TEM) to examine radiation-induced effects on mitochondrial degeneration in the hippocampus. We analyzed mitochondria from naïve (non-irradiated mice), FB treated mice irradiated at 10.0 Gy, and BBT-059 treated mice irradiated at 10.5, 11.5 and 12.0 Gy after 6 and 12 months post-TBI. Mitochondria were evaluated with respect to ultrastructure: the rupture of the mitochondrial outer membrane, swelling of mitochondria, and the inner matrix of the mitochondria. Representative examples of ultrastructural changes for the different treatment groups are shown in Figure 1A. At six and twelve-month post-TBI, the ultrastructural study revealed that the number of degenerating mitochondria was higher in groups that were irradiated at higher radiation doses. At 6 months post-TBI the number of degenerating mitochondria were significantly higher in the 12 Gy BBT-059 treated group as compared to naïve (*p* = 0.0001), however, there was no significant difference in the 10 Gy FB group compared to naïve or in the 10.5 Gy, BBT-059 and the 11.5 Gy, BBT-059 treated groups compared to naïve (Figure 1). At 12 months post-TBI, the number of degenerating mitochondria were significantly higher in the 11.5 Gy BBT-059 group compared to naïve (*p* = 0.001); however the number of degenerating mitochondria were not significantly different between the 10.0 Gy FB treated group and the 10.5 Gy BBT-059 treated groups compared to naïve (Figure 1B). BBT-059 treated animals irradiated at 12 Gy did not survive beyond 6 months post-TBI. However, autophagosome envelops the mitochondria were also counted from electron microscopy pictures and a trend was observed with the radiation doses, however, no significant difference was observed between different groups (Figure 1C). The Bar graphs in the figure shows the average number from twelve pictures from different regions of three independent animals (Scale bar 500 nm) at 6 and 12 months.

### Western Blot and IHC Analysis of Autophagy-Related Proteins

**Figure 2:**
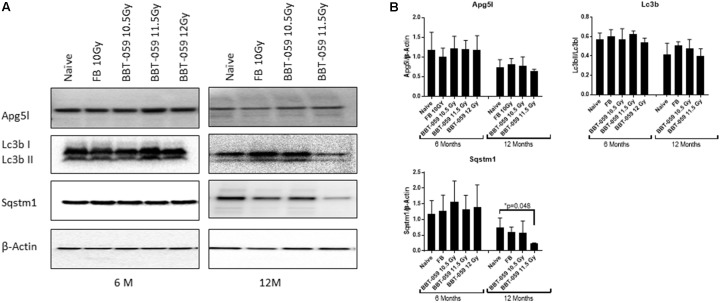
Western blot analysis showing a decrease in autophagy marker after 12 months in the brain.** (A)** Brains from animals treated with formulation buffer 10 Gy and BBT-059 were collected at 6 and 12 months post-TBI. The figure shows the representative images of western blots from the brain at 6 and 12 months of naive, 10 Gy irradiated pre-treated with formulation buffer (FB 10 Gy), 10.5, 11.5, and 12 Gy irradiated and pre-treated with BBT-059. **(B)** The bar graph shows the quantification of the ratio of protein of interest and its respective β-Actin control from three independent animals. After 6 months, no significant difference was observed in different groups, however, after 12 months the expression of Sqstm1 protein was significantly less in 11.5 Gy BBT-059 group as compared to naïve (*p* = 0.048). Mann–Whitney *U* test was applied for analysis. *p < 0.05.

**Figure 3:**
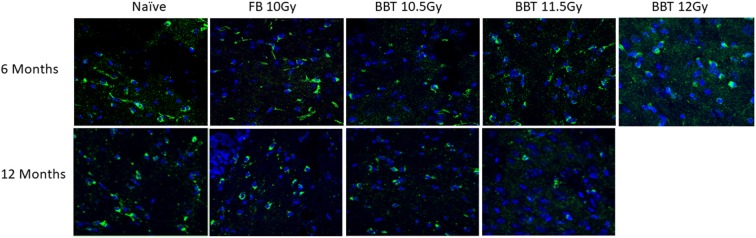
Higher radiation dose leads to autophagy as delayed effects. Brain from naive and animals treated with formulation buffer and BBT-059 were collected at 6 and 12 months post-TBI. The figure shows the representative images of Bcl2 immunohistochemistry (IHC) in brains at 6 and 12 months from naive, 10 Gy irradiated pre-treated with formulation buffer (FB 10 Gy), 10.5, 11.5, and 12 Gy irradiated and pre-treated with BBT-059. At 6 months post-TBI we did not observe any difference between different groups for Bcl2 expression, however, after 12 months post-TBI, a lower expression was observed in the group administered BBT-059 24 h prior to TBI at 11.5 Gy compared to all other groups.

We further investigated changes in levels of autophagy-related biomarkers in brain sections by western blot. Antibodies against Apg5l, Lc3b (Lc3bI and Lc3bII) and Sqstm1 proteins were used for western blots of a part of left-brain lysates collected at 6 and 12 months post-TBI (Figure 2). β-actin was used as an internal control protein. At 6 months post-TBI we did not observe any significant differences between different groups for Apg5l, Lc3b (Lc3b-II/Lc3b-I ratio) and Sqstm1 proteins. At 12 months post-TBI, a statistically significant difference was observed in Sqstm1 protein levels. The Sqstm1 protein expression was reduced in the group administered BBT-059 24 h prior to TBI at 11.5 Gy compared to the naïve group (*p* = 0.048). The same kind of results was observed with Bcl2 IHC. At 6 months post-TBI we did not observe any difference between different groups for Bcl2 expression, however, after 12 months post-TBI, a lower expression was observed in Bcl2 protein. Bcl2 expression was reduced in the group administered BBT-059 24 h prior to TBI at 11.5 Gy compared to all other groups (Figure 3).

## Discussion

Our previous data on BBT-059 suggest that it protects the hematopoietic system as well as inhibits the upregulation of hematopoietic cytokines, and this is a promising radiation countermeasure for medical management which can be used in a preventive manner as and as a mitigator for radiation injury (Kumar et al., 2018). Our unpublished data showed excellent enhancement in a 30-day survival study following the administration of BBT-059 with a high DRF of 1.28. An evaluation of DRF for 30-day survival (LD50/30) allows for a comparison of medical countermeasures that protect against radiation-induced hematopoietic mortality (Wang et al., 2019) in the mouse model. When surviving animals were monitored up to 12 months post-TBI after the DRF study, bone marrow progenitor cells and sternal megakaryocytes showed recovery in BBT-059 treated groups compared to naïve on day 30 and 6 months post-TBI (data in the communication). In this study, electron microscopy data on brain sections demonstrated significant DEARE in mice exposed to TBI at lethal doses of 11.5 and 12 Gy.

Epidemiological studies on human populations have demonstrated the late-onset effects of exposure to IR. Population studies from Hiroshima documented various neurodegenerative, cancer, and cerebrovascular diseases as delayed effects in survivors. They documented that depending upon the density of exposure and age of exposure to radiation from the atomic bombing, there was a several-fold increase in the risk of certain diseases with age (Douple et al., 2011; Jordan, 2016; Sharma et al., 2018). Previously, it was reported that mitochondria are the target organelles for IR. Radiation can cause direct damage to DNA and proteins or indirect damage by producing excessive ROS (Kam and Banati, 2013). Compromised mitochondrial function, endoplasmic reticulum (ER) stress, and protein misfolding can be caused by radiation-induced oxidative stress. For the normal brain function mitophagy and oxidative phosphorylation, mitochondrial fusion and fission are very important (Fuhrmann and Brüne, 2017). Mitochondrial degeneration can cause cell body death (Jordán et al., 2003) due to progressive loss of axonal length (Saxton and Hollenbeck, 2012). Proper mitochondrial function is very important for maintaining homeostasis, cellular integrity, and neurons in the brain (Jing et al., 2012). Moreover, the mitochondria need to be transported along the axons to sites of high energy demand to carry out cellular functions normally.

In this study, we demonstrate that high doses of IR change the dynamics of mitochondria in the hippocampus and observed a significantly higher number of degenerating mitochondria in the group exposed to higher doses of radiation (12 Gy); however, at lower but still lethal radiation doses BBT-059 treatment appears to protect the hippocampus from mitochondrial degeneration, as indicated by no significant difference in the number of degenerating mitochondria as compared to naive. The 12 Gy, BBT-059 treated group did not survive up to 12 months therefore after 12 months 11.5 Gy was the highest irradiated group, and they had a significantly higher number of mitochondria degenerated as compared to the other groups. This indicates that higher radiation doses increased the mitochondrial degeneration as compared to lower radiation doses up to 11.5 Gy. Since due to *supra* lethal doses we were not able to analyze 10.5 Gy, 11.5 Gy and 12 Gy FB treated mice, therefore, we cannot say whether BBT-059 provides partial protection against mitochondrial degeneration at these radiation doses. Mitochondrial function is important for the active maintenance of mitochondria in a constant supply of energy for the functioning of neurons and synaptic plasticity. In addition, due to dysfunctional mitochondria, there is a direct effect on anterograde and retrograde trafficking, which eventually affects neuronal trafficking across the neurons. Damaged mitochondria are removed through autophagy, which is a natural self-degradative process by which cells eliminate damaged organelles and misfolded proteins. Autophagy has recently been identified as an effector of senescence (Young and Narita, 2010). Our results indicated that autophagy markers (Apg5l, Lc3b and Sqstm1) did not show any changes in their expression at the six-month post-TBI time point, though after 12 months the 11.5 Gy, BBT-059 group showed a significant decrease in Sqstm1 expression compared to all other groups. Sqstm1 is the main regulator of the autophagic pathway that directs ubiquitinated cargoes to autophagosomes for degradation (Sebastiani et al., 2017). Autophagy is an evolutionarily conserved process, morphologically it is involved in the formation of double-membrane bound vesicles known as autophagosomes or autophagic vacuoles that degrade, thus recycling proteins and cellular organelles by fusion with lysosomes (Yang and Klionsky, 2009). Galindo-Moreno et al. (2017) demonstrated that after treatment with any DNA-damaging agents, Sqstm1 is degraded *via* the lysosomal pathway. Our results for Sqstm1 were supported by the results of IHC for Bcl2 protein. Bcl2 expression did not show any change in their expression after 6 months post-TBI time point, however after 12 months the 11.5 Gy, BBT-059 group showed a decrease in Bcl2 expression compared to all other groups. Bcl2 family of proteins is the hallmark of apoptosis regulation. BCL2 is permanently found in membranes. Bcl-2 is a pro-survival protein that inhibits apoptosis or autophagy. It seems to inhibit apoptosis by the protection of mitochondrial membrane integrity as its hydrophobic carboxyl-terminal domain is linked to the outer membrane. It is already known that Bcl2 deactivates BAX and other pro-apoptotic proteins, thereby preventing apoptosis. Several initiator caspases like caspase-2 that act upstream or independently of cytochrome c release from mitochondria might also be regulated by BCL2. Furthermore, BCL2 directly blocks cytochrome-c release and so prevents caspase-9 activation (Tzifi et al., 2012). It is also likely that the anti-apoptotic action of Bcl2 is changed to a proapoptotic one when protein is cleaved by caspases after initiation of apoptosis (Bellows et al., 2000) and can dysregulate the autophagy process. Although we did not detect the LC3-II change in immunoblots, this could be because the excess autophagosomes rapidly fuse with lysosomes in the brain thus preventing LC3-II accumulation. However, there is also the possibility of additional mechanisms besides LC3-related autophagy to activate lysosomes and digest the cytoplasm of damaged cells (Mizushima, 2004).

Several studies in different human diseases have been identified as the importance of dysregulated autophagy. In healthy neurons, autophagy constitutively occurs at low levels. Depending on the condition, autophagy may serve as a pro-death or pro-survival mechanism (Rubinsztein et al., 2005). Dysregulation autophagy or mitophagy has been stated to happen in numerous neurodegenerative processes like cerebral ischemia (Xu et al., 2016), post-traumatic stress disorder (Zheng et al., 2017), AD (Kuusisto et al., 2002) or PD (Wang et al., 2016). Previously it was revealed that autophagy flux regulated by glycogen synthase (GS) in the active state and helps the aggregate load of the cell to clear (Rai et al., 2018). Excessive autophagy is induced in neuron due to overexpression of glycogen synthesizing proteins and that the excessive autophagy is the cause of the death of many cellular organelles like mitochondria. Other studies, also suggest that enhanced autophagy may contribute to neuronal death in various pathological conditions including cerebral ischemia (Shi et al., 2012). Still, arguments exist whether increased autophagy activities lead to autophagy neuronal death (Wong and Cuervo, 2010). Metabolic energy regulation may be affected by mitochondrial dysfunction and the excess autophagy can lead to damage in cellular structure. However, in our study, the animals treated with BBT-059 were survived long term after exposure to supra-lethal radiation doses and the mitochondrial defects in the hippocampus were radiation dose-dependent. The main limitation of this study was less number of animals in each group. A better understanding of the molecular pathways involved in mitochondrial deterioration could lead to the identification of novel therapeutic targets for improved treatment of mitochondrial damage due to exposure to IR.

## Data Availability Statement

The raw data supporting the conclusions of this article will be made available by the authors, without undue reservation, to any qualified researcher.

## Ethics Statement

The animal study was reviewed and approved by the institutional animal care and use committee at the Armed Forces Radiobiology Research Institute (AFRRI).

## Author Contributions

NS: conception, design of the work, acquisition, analysis, interpretation of data, drafting the manuscript and critical reviewing. SS, VK, SB, SA, GH-H, CF and GC: acquisition of data and critical reviewing. SG: conception, design of the work, drafting the manuscript, and critical reviewing. All authors gave final approval of the version to be published.

## Conflict of Interest

None of the following authors NS, SS, VK, SB, SA, GH-H, and SG have a financial interest in any commercial product, service, or organization providing financial support for this research. CF and GC are employees of Bolder Biotechnology and have a financial interest in the company.

## References

[B1] Bellows D. S. Chau B. N. Lee P. Lazebnik Y. Burns W. H. Hardwick J. M. (2000). Antiapoptotic herpesvirus Bcl-2 homologs escape caspase-mediated conversion to proapoptotic proteins. J. Virol. 74, 5024–5031. 10.1128/jvi.74.11.5024-5031.2000 10799576 PMC110854

[B2] Cadenas E. Davies K. J. (2000). Mitochondrial free radical generation, oxidative stress, and aging. Free Radic. Biol. Med. 29, 222–230. 10.1016/s0891-5849(00)00317-8 11035250

[B3] Douple E. B. Mabuchi K. Cullings H. M. Preston D. L. Kodama K. Shimizu Y.. (2011). Long-term radiation-related health effects in a unique human population: lessons learned from the atomic bomb survivors of Hiroshima and Nagasaki. Disaster Med Public Health Prep. 5, S122–S133. 10.1001/dmp.2011.21 21402804 PMC3907953

[B4] Fuhrmann D. C. Brüne B. (2017). Mitochondrial composition and function under the control of hypoxia. Redox Biol. 12, 208–215. 10.1016/j.redox.2017.02.012 28259101 PMC5333533

[B5] Galindo-Moreno M. Giráldez S. Sáez C. Japón M. A. Tortolero M. Romero F. (2017). Both p62/SQSTM1-HDAC6-dependent autophagy and the aggresome pathway mediate CDK1 degradation in human breast cancer. Sci. Rep. 7:10078. 10.1038/s41598-017-10506-8 28855742 PMC5577189

[B6] Harada K. H. Niisoe T. Imanaka M. Takahashi T. Amako K. Fujii Y.. (2014). Radiation dose rates now and in the future for residents neighboring restricted areas of the Fukushima Daiichi Nuclear Power Plant. Proc. Natl. Acad. Sci. U S A 111, E914–E923. 10.1073/pnas.1315684111 24567380 PMC3956155

[B7] Hauer-Jensen M. (2014). Toward development of interleukin-11 as a medical countermeasure for use in radiological/nuclear emergencies. Dig. Dis. Sci. 59, 1349–1351. 10.1007/s10620-014-3074-x 24591015 PMC4071113

[B8] Jing C. H. Wang L. Liu P. P. Wu C. Ruan D. Chen G. (2012). Autophagy activation is associated with neuroprotection against apoptosis *via* a mitochondrial pathway in a rat model of subarachnoid hemorrhage. Neuroscience 213, 144–153. 10.1016/j.neuroscience.2012.03.055 22521819

[B9] Jordan B. R. (2016). The hiroshima/nagasaki survivor studies: discrepancies between results and general perception. Genetics 203, 1505–1512. 10.1534/genetics.116.191759 27516613 PMC4981260

[B10] Jordán J. Ceña V. Prehn J. H. (2003). Mitochondrial control of neuron death and its role in neurodegenerative disorders. J. Physiol. Biochem. 59, 129–141. 10.1007/bf03179878 14649878

[B11] Kam W. W. Banati R. B. (2013). Effects of ionizing radiation on mitochondria. Free Radic. Biol. Med. 65, 607–619. 10.1016/j.freeradbiomed.2013.07.024 23892359

[B12] Kumar V. P. Biswas S. Sharma N. K. Stone S. Fam C. M. Cox G. N.. (2018). PEGylated IL-11 (BBT-059): a novel radiation countermeasure for hematopoietic acute radiation syndrome. Health Phys. 115, 65–76. 10.1097/hp.0000000000000841 29787432 PMC5967654

[B13] Kuusisto E. Salminen A. Alafuzoff I. (2002). Early accumulation of p62 in neurofibrillary tangles in Alzheimer’s disease: possible role in tangle formation. Neuropathol. Appl. Neurobiol. 28, 228–237. 10.1046/j.1365-2990.2002.00394.x 12060347

[B14] Leach J. K. Van Tuyle G. Lin P. S. Schmidt-Ullrich R. Mikkelsen R. B. (2001). Ionizing radiation-induced, mitochondria-dependent generation of reactive oxygen/nitrogen. Cancer Res. 61, 3894–3901. 11358802

[B15] Lee H. T. Park S. W. Kim M. Ham A. Anderson L. J. Brown K. M.. (2012). Interleukin-11 protects against renal ischemia and reperfusion injury. Am. J. Physiol. Renal Physiol. 303, F1216–E1224. 10.1152/ajprenal.00220.2012 22859402 PMC3469680

[B16] Lezi E. Swerdlow R. H. (2012). Mitochondria in neurodegeneration. Adv. Exp. Med. Biol. 942, 269–286. 10.1007/978-94-007-2869-1_12 22399427 PMC3618469

[B17] Lou G. Palikaras K. Lautrup S. Scheibye-Knudsen M. Tavernarakis N. Fang E. F. (2019). Mitophagy and neuroprotection. Trends Mol. Med. [Epub ahead of print]. 10.1016/j.molmed.2019.07.002 31375365

[B18] Mizushima N. (2004). Methods for monitoring autophagy. Int. J. Biochem. Cell Biol. 36, 2491–2502. 10.1016/j.biocel.2004.02.005 15325587

[B19] Paul S. R. Bennett F. Calvetti J. A. Kelleher K. Wood C. R. O’Hara R. M.. (1990). Molecular cloning of a cDNA encoding interleukin 11, a stromal cell-derived lymphopoietic and hematopoietic cytokine. Proc. Natl. Acad. Sci. U S A 87, 7512–7516. 10.1073/pnas.87.19.7512 2145578 PMC54777

[B20] Plett P. A. Chua H. L. Sampson C. H. Katz B. P. Fam C. M. Anderson L. J.. (2014). PEGylated G-CSF (BBT-015), GM-CSF (BBT-007), and IL-11 (BBT-059) analogs enhance survival and hematopoietic cell recovery in a mouse model of the hematopoietic syndrome of the acute radiation syndrome. Health Phys. 106, 7–20. 10.1097/hp.0b013e3182a4dd4e 24276546 PMC3843149

[B21] Pospisil P. Kazda T. Bulik M. Dobiaskova M. Burkon P. Hynkova L.. (2015). Hippocampal proton MR spectroscopy as a novel approach in the assessment of radiation injury and the correlation to neurocognitive function impairment: initial experiences. Radiat. Oncol. 10:211. 10.1186/s13014-015-0518-1 26474857 PMC4609038

[B22] Rai A. Singh P. K. Singh V. Kumar V. Mishra R. Thakur A. K.. (2018). Glycogen synthase protects neurons from cytotoxicity of mutant huntingtin by enhancing the autophagy flux. Cell Death Dis. 9:201. 10.1038/s41419-017-0190-5 29422655 PMC5833817

[B23] Rubinsztein D. C. DiFiglia M. Heintz N. Nixon R. A. Qin Z. H. Ravikumar B.. (2005). Autophagy and its possible roles in nervous system diseases, damage and repair. Autophagy 1, 11–22. 10.4161/auto.1.1.1513 16874045

[B24] Rubinsztein D. C. Mariño G. Kroemer G. (2011). Autophagy and aging. Cell 146, 682–695. 10.1016/j.cell.2011.07.030 21884931

[B25] Saxton W. M. Hollenbeck P. J. (2012). The axonal transport of mitochondria. J. Cell Sci. 125, 2095–2104. 10.1242/jcs.053850 22619228 PMC3656622

[B26] Sebastiani A. Gölz C. Sebastiani P. G. Bobkiewicz W. Behl C. Mittmann T.. (2017). Sequestosome 1 deficiency delays, but does not prevent brain damage formation following acute brain injury in adult mice. Front. Neurosci. 11:678. 10.3389/fnins.2017.00678 29311767 PMC5742218

[B27] Sharma N. K. Sharma R. Mathur D. Sharad S. Minhas G. Bhatia K.. (2018). Role of ionizing radiation in neurodegenerative diseases. Front. Aging Neurosci. 10:134. 10.3389/fnagi.2018.00134 29867445 PMC5963202

[B28] Shi R. Weng J. Zhao L. Li X. M. Gao T. M. Kong J. (2012). Excessive autophagy contributes to neuron death in cerebral ischemia. CNS Neurosci. Ther. 18, 250–260. 10.1111/j.1755-5949.2012.00295.x 22449108 PMC6493486

[B29] Shimura T. Kunugita N. (2016). Mitochondrial reactive oxygen species-mediated genomic instability in low-dose irradiated human cells through nuclear retention of cyclin D1. Cell Cycle 15, 1410–1414. 10.1080/15384101.2016.1170271 27078622 PMC4934070

[B30] Tzifi F. Economopoulou C. Gourgiotis D. Ardavanis A. Papageorgiou S. Scorilas A. (2012). The role of BCL2 family of apoptosis regulator proteins in acute and chronic leukemias. Adv. Hematol. 2012:524308. 10.1155/2012/524308 21941553 PMC3173728

[B31] Valentin J. (2005). Low-dose extrapolation of radiation-related cancer risk. Ann. ICRP 35, 1–140. 10.1016/j.icrp.2005.11.002 16782497

[B33] Wang X. (2001). The expanding role of mitochondria in apoptosis. Genes Dev. 15, 2922–2933. 11711427

[B32] Wang B. Cai Z. Tao K. Zeng W. Lu F. Yang R.. (2016). Essential control of mitochondrial morphology and function by chaperone-mediated autophagy through degradation of PARK7. Autophagy 12, 1215–1228. 10.1080/15548627.2016.1179401 27171370 PMC4968227

[B34] Wang Y. Liu N. Lu B. (2019). Mechanisms and roles of mitophagy in neurodegenerative diseases. CNS Neurosci. Ther. 25, 859–875. 10.1111/cns.13140 31050206 PMC6566062

[B35] Williams J. P. Brown S. L. Georges G. E. Hauer-Jensen M. Hill R. P. Huser A. K.. (2010). Animal models for medical countermeasures to radiation exposure. Radiat. Res. 173, 557–578. 10.1667/RR1880.1 20334528 PMC3021126

[B36] Wong E. Cuervo A. M. (2010). Autophagy gone awry in neurodegenerative diseases. Nat. Neurosci. 13, 805–811. 10.1038/nn.2575 20581817 PMC4038747

[B37] Xu Y. Tian Y. Tian Y. Li X. Zhao P. (2016). Autophagy activation involved in hypoxic-ischemic brain injury induces cognitive and memory impairment in neonatal rats. J. Neurochem. 139, 795–805. 10.1111/jnc.13851 27659442

[B38] Yang Z. Klionsky D. J. (2009). An overview of the molecular mechanism of autophagy. Curr. Top. Microbiol. Immunol. 335, 1–32. 10.1007/978-3-642-00302-8_1 19802558 PMC2832191

[B39] Young A. R. Narita M. (2010). Connecting autophagy to senescence in pathophysiology. Curr. Opin. Cell Biol. 22, 234–240. 10.1016/j.ceb.2009.12.005 20045302

[B40] Zheng S. Han F. Shi Y. Wen L. Han D. (2017). Single-prolonged-stress-induced changes in autophagy-related proteins beclin-1, LC3, and p62 in the medial prefrontal cortex of rats with post-traumatic stress disorder. J. Mol. Neurosci. 62, 43–54. 10.1007/s12031-017-0909-x 28341893

